# Comparing Ultrasound-based Diaphragmatic Excursion with Rapid Shallow Breathing Index as a Weaning Predictor

**DOI:** 10.7759/cureus.3710

**Published:** 2018-12-10

**Authors:** Muhammad Tariq Khan, Kamran Munawar, Syed Waqar Hussain, Aayesha Qadeer, Muhammad Luqman Saeed, Zahid Siddique Shad, Muhammad Shoaib Safdar Qureshi, Azmat Abdullah

**Affiliations:** 1 Pulmonology, Shifa International Hospital, Islamabad, PAK; 2 Internal Medicine, Shifa College of Medicine, Islamabad, PAK; 3 Internal Medicine, Khan Research Laboratories Hospital, Islamabad, PAK; 4 Internal Medicine, Shifa International Hospital, Islamabad, PAK; 5 Internal Medicine, Shifa International Hospital, Islamabad, USA

**Keywords:** intensive care units (icu), intensivist, clinical practitioners, extubation, weaning predictor

## Abstract

Background

A challenging task in the intensive care unit is weaning intubated patients from mechanical ventilation. The most commonly used weaning parameter, the rapid shallow breathing index (RSBI), gives thorough guidance on extubation timing with spontaneous breathing trials. Diaphragm plays vital role in tidal volume generation. The main objective of the study was to compare ultrasound-based diaphragmatic excursion (DE) with RSBI as weaning predictors.

Methods

We conducted an observational prospective cohort study on patients on mechanical ventilation. During a spontaneous breathing trial (SBT) we simultaneously evaluated right hemidiaphragm excursion by using M-mode ultrasonography as well as the RSBI. To be included, patients must have been on mechanical ventilation for longer than 48 hours, have no excessive tracheobronchial secretions, and their underlying critical illness (for which they were intubated) must be resolved. Patients younger than 14 years, patients with neuromuscular disorder, patients with pneumothorax, and patients with cervical spine injury were excluded from the study. We analyzed the data to determine the significance of DE and RSBI.

Results

A total of 90 patients were included in our study; 54 (60%) were men, and 36 (40%) were women. The average age of all the participants was 55 ± 16 years (range, 19 to 83 years). Sixty-two patients (68.9%) were successfully weaned. The mean DE was 1.44 ± 0.26 cm, and the mean RSBI was 56.88 ± 8.30 in all patients. Successful weaning patients had a mean DE of 1.51 ± 0.26 cm and a mean RSBI of 54.05 ± 7.00. The greater the DE value, the greater the weaning success rate, and the lesser the RSBI value, the greater the weaning success rate. The area under the receiver operator curve for DE and RSBI was 0.795 and 0.815, respectively (p < 0.0001).

Conclusion

RSBI is an optimized clinical predictor in classifying weaning outcomes for intubated patients, but DE is also helpful in extubation assurance and reintubation prevention.

## Introduction

For any intensivist working in an intensive care unit (ICU), weaning patients from mechanical ventilation is a challenging task because unnecessary delay can lead to further complications. For years, studies have tried to address the difficulties of weaning, but getting patients to regain spontaneous breathing remains a dilemma for clinician and practitioners. Approximately 20% of the intubated patients present with difficulty in extubation and weaning, despite established weaning criteria [1]. Numerous indices have been devised to assess a patient’s ability to regain spontaneous breathing during weaning such as maximum inspiratory pressure, minute ventilation, breathing frequency (rate), rapid shallow breathing index (RSBI, i.e., respiratory frequency per tidal volume), tracheal airway occlusion pressure, oxygen pressure index, and gastric pressure monitoring [2]. Yang and Tobin reported RSBI was the most accurate weaning predictor [3]. Ely et al. reported that decisions based on certain predictors related to weaning often resulted in poor outcomes [4]. Therefore, regularly assessing breathing frequency and negative inspiratory force may contribute to successful extubation. A recent weaning parameter, D-RSBI, provides thorough guidance in extubation timing with spontaneous breathing trials (SBT) [5]. The diaphragm is a fundamental respiratory muscle whose dysfunction may be very common in patients undergoing mechanical ventilation. Impaired diaphragmatic function may lead to difficulty in weaning [6]. Different diagnostic tools can uncover diaphragm dysfunction like fluoroscopy, phrenic nerve conduction study, percussion method, and trans-diaphragmatic pressure measurements [7]. Currently, ultrasound is a favorite modality for evaluating diaphragm dysfunction [8]. The main objective of this study was to assess if ultrasound-based diaphragmatic excursion (DE) is helpful with RSBI as weaning predictors.

## Materials and methods

We conducted an observational prospective cohort study consisting of 90 patients on mechanical ventilation in the medical intensive care department of Shifa International Hospital in Islamabad, Pakistan. The sample size was calculated by considering the margin of error as 5%, 95% confidence interval and prevalence of 67% [5]. The duration of the study was one year starting from July 2017. Patients were excluded if they were younger than 14 years old, had a neuromuscular disorder, pneumothorax or cervical spine injuries. To be included, patients must have been on mechanical ventilation for longer than 48 hours, have no excessive tracheobronchial secretions, and their underlying critical illness (their reason for intubation) must have been resolved. All study participants were alert, cooperative, and hemodynamically stable. The arterial oxygen saturation was above 90%, and fraction of inspired oxygen (FiO2) was at least 30%.

All patients or their legal guardians provided informed consent for participation in this study. The data were collected via a validated questionnaire and diagnostic assessments. Demographic details, medical history, and clinical presentations were entered in a proforma. The diaphragm ultrasound was performed at the time of the SBT after at least 48 hours of mechanical ventilation. Bedside ultrasound was performed by two critical care medicine fellows in the ICU trained in lung and abdominal ultrasound. Interobserver variability was 1 mm to 3 mm. A curvilinear probe (3.5 to 5 MHz) was placed at the right hypochondrial area, and the movement of the diaphragm was observed via the B mode as the diaphragm moved cranially to caudally with respiration. Then, M mode was used to measure the diaphragmatic excursion in centimeters. Other diagnostic values like RSBI were also noted. After the extubation, all patients were monitored for failed or successful weaning. Weaning was considered successful if the patient did not require noninvasive or invasive ventilation within 48 hours of extubation. Re-instituting mechanical ventilatory support occurred if one of the following criteria was met: sweating, anxiety, agitation, deterioration in neurological status, abdominal paradox, usage of accessory muscles, breathing rate exceeding 30/minute, arterial carbon dioxide > 55 mmHg, pH < 7.25, arterial partial pressure of oxygen < 70 mmHg at FIO2 of > 0.5, systolic blood pressure > 180 mmHg or < 90 mmHg, heart rate > 140 beats/minute, a sustained 20% increase or decrease in heart rate, and unstable hemodynamics.

Statistical analysis

All the data collected were analyzed using SPSS Statistics for Windows, Version 21.0 (IBM Corp., Armonk, NY). Descriptive statistics were applied by calculating mean and standard deviation. Frequency distribution and percentages were performed for all qualitative variables. P values less than 0.05 were considered statistically significant in all inferential statistics. The differences of continuous variables between the subgroups for the independent variable were assessed by non-parametric tests. We used the Kruskal–Wallis test to determine the significance of DE and RSBI as indicative parameters. The area under the receiver operator curve (AUROC) was applied to evaluate the diagnostic accuracy of DE and RSBI.

## Results

We recruited a total of 90 patients for this study; 54 (60%) were men, and 36 (40%) were women. The average age of the participants was 55 ± 16 years (range, 19 to 83 years). Most patients (63%) required mechanical ventilation due to respiratory problems. No surgical patients were included in the study.

**Figure 1:**
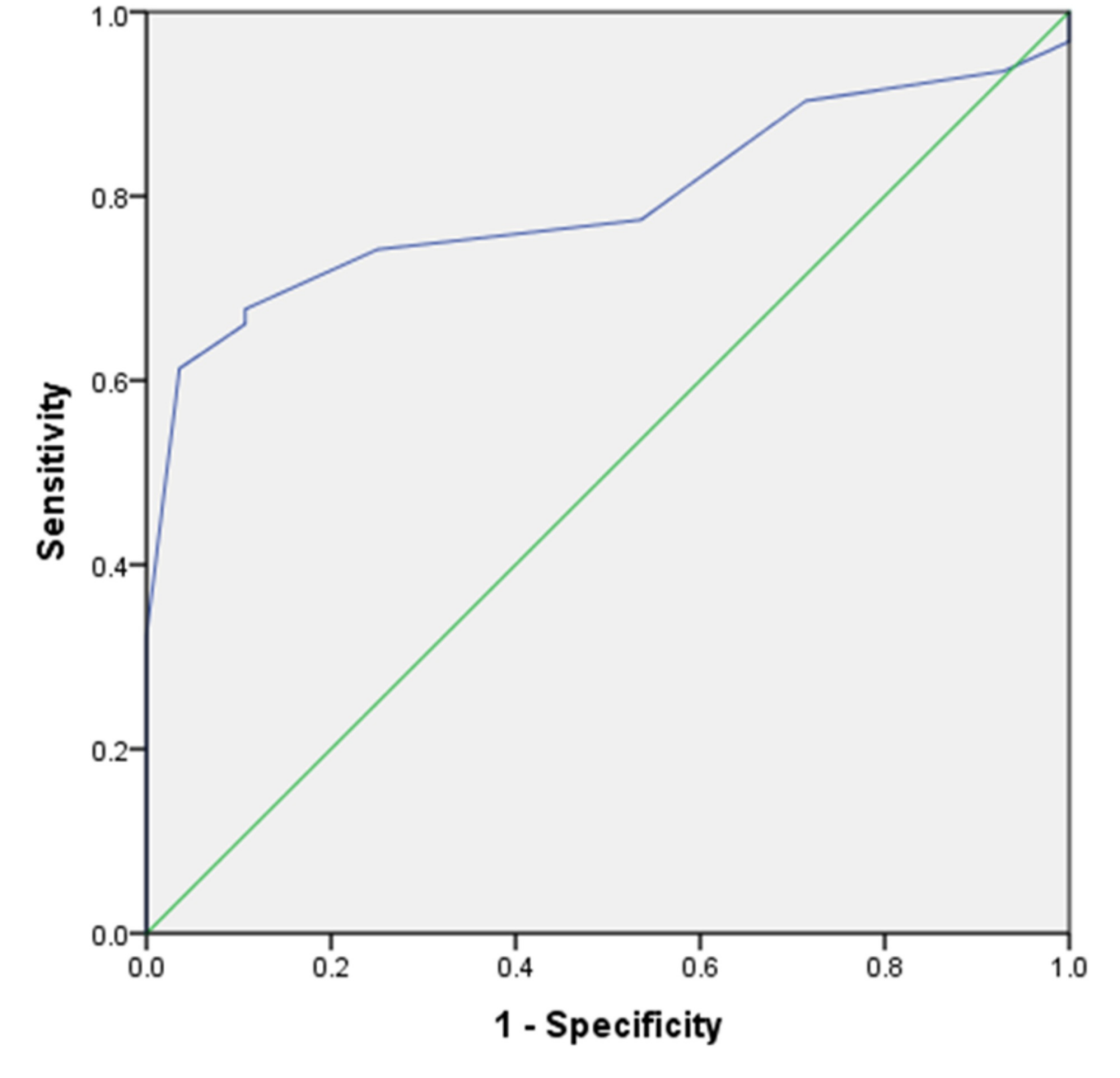
ROC curve revealing cut-off point for sensitivity and specificity of diaphragmatic excursion. AUROC = 0.795. AUROC: Area under the receiver operator curve.

**Figure 2:**
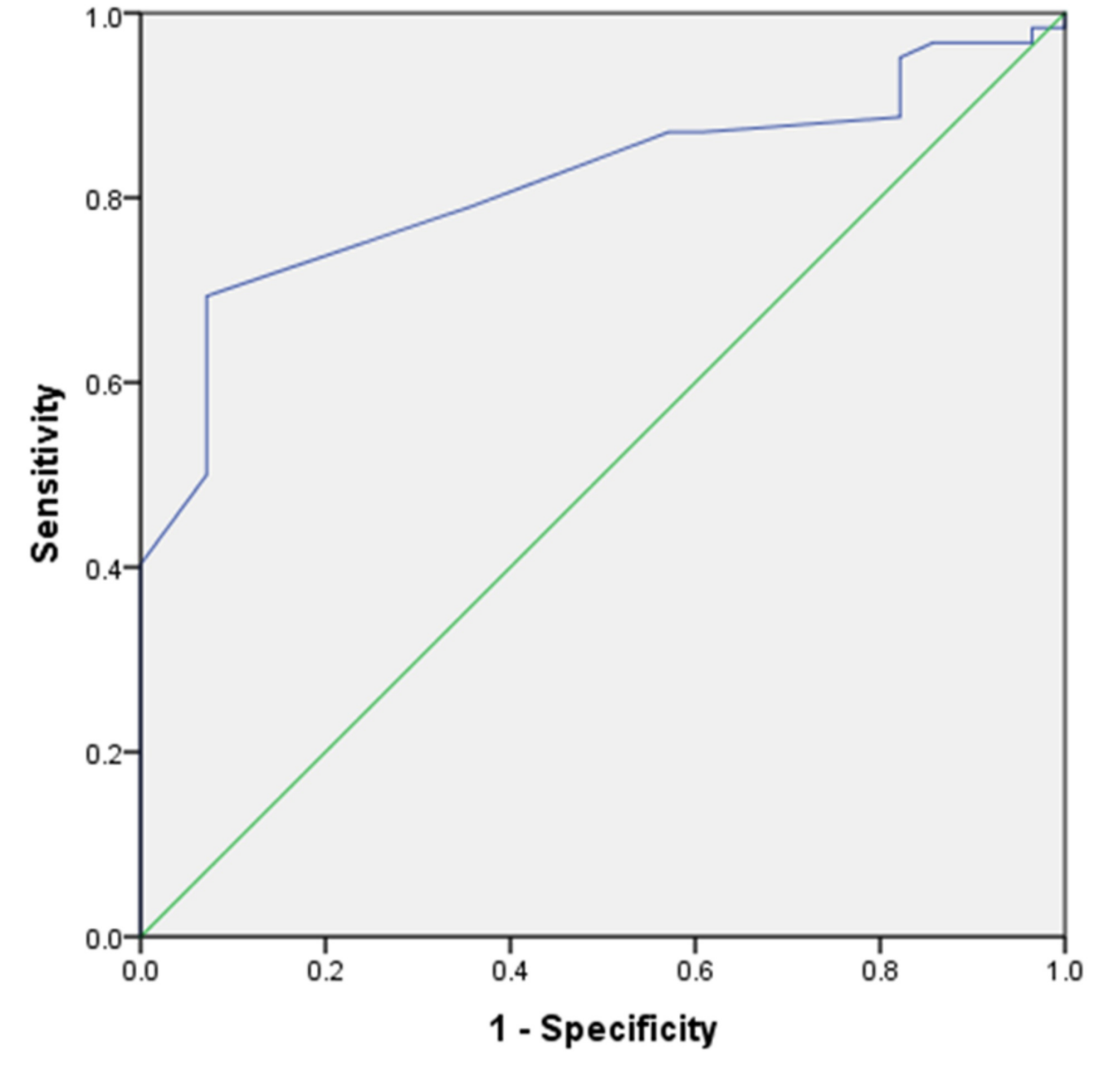
ROC curve revealing cut-off point for sensitivity and specificity of RSBI. AUROC = 0.815. AUROC: Area under the receiver operator curve; RSBI: Rapid shallow breathing index.

**Figure 3:**
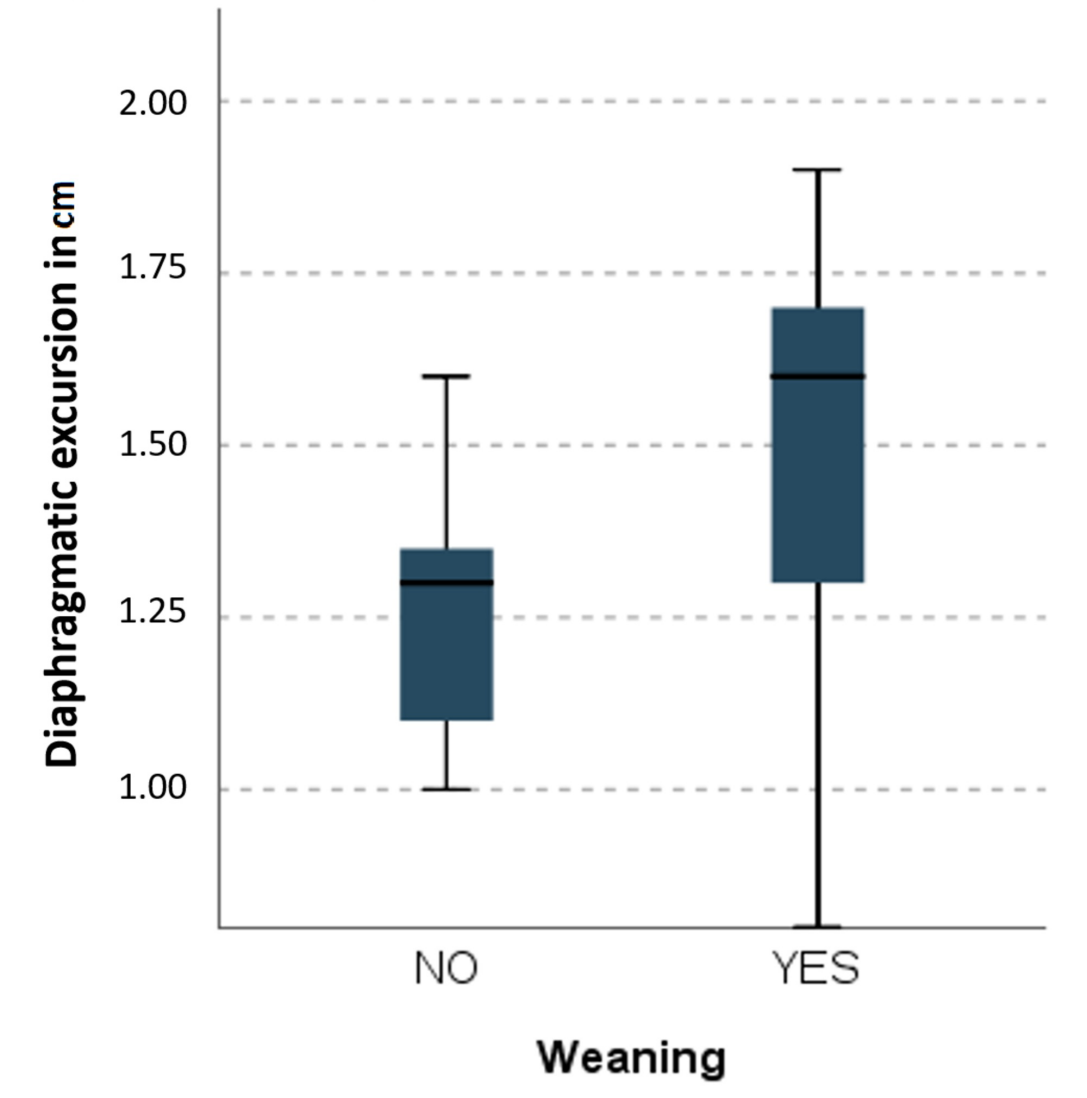
Kruskal–Wallis test of weaning using diaphragmatic excursion as a predictor.

**Figure 4:**
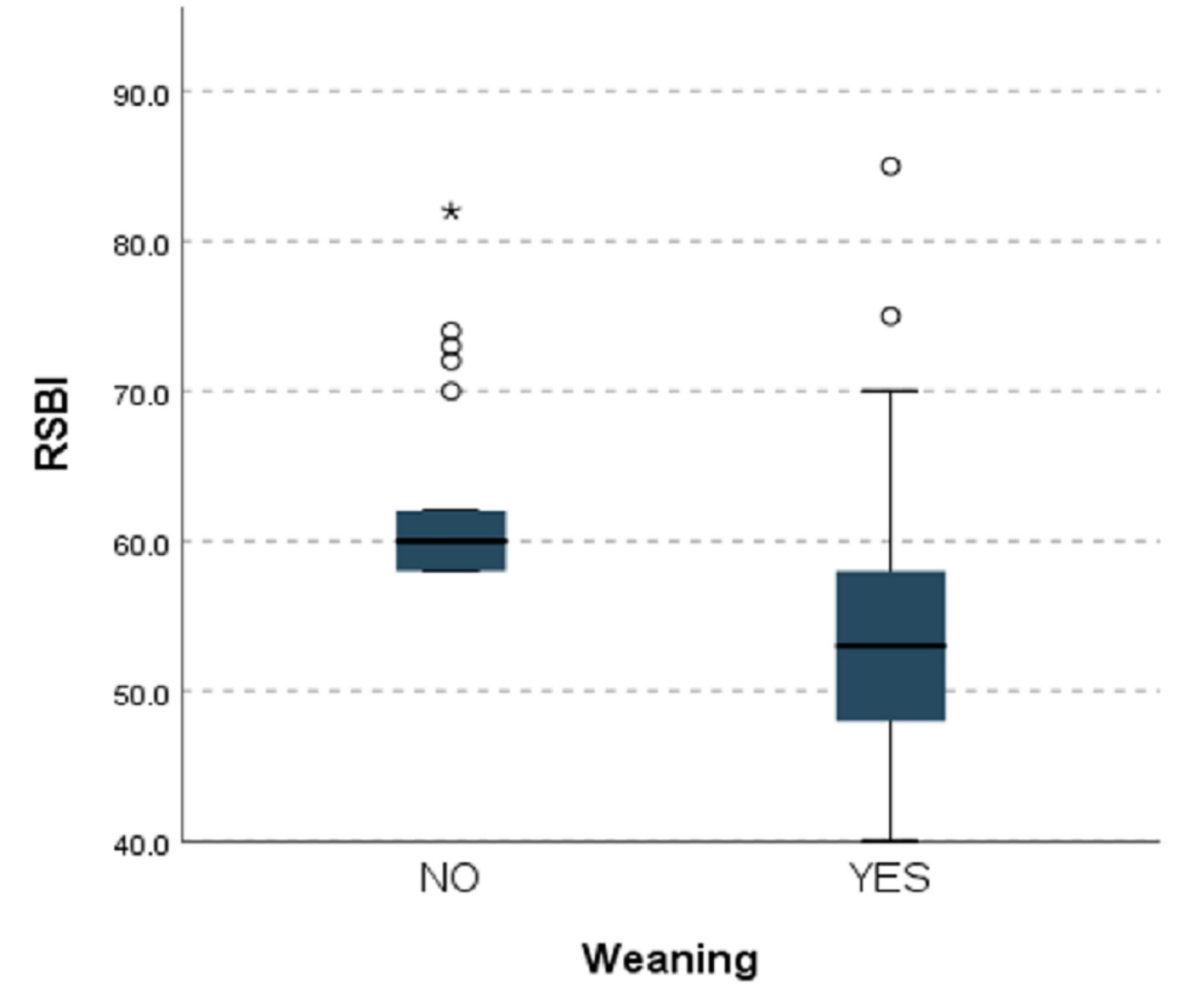
Kruskal–Wallis test of weaning using RSBI as a predictor. RSBI: Rapid shallow breathing index

Sixty-two of 90 patients were successfully weaned (68.9%). RSBI and DE were analyzed for all patients. The mean DE was 1.44 ± 0.26 cm, and the mean RSBI was 56.88 ± 8.30 for all patients. The difference between the failed and successful groups was statistically significant for both DE (p < 0.0001) and RSBI (p < 0.0001; Table 1). DE had an AUROC of 0.795 (95% confidence interval [CI]: 0.705 to 0.885; p < 0.001), and RSBI had an AUROC of 0.815 (95% CI: 0.728 to 0.902; p < 0.001). Our RSBI cutoff value of 59 showed 79% sensitivity and 64% specificity. The DE cutoff value of 1.35 cm had 74% sensitivity and 75% specificity (Figures 1, 2). The greater the DE value, the greater the weaning success rate, and the lesser the RSBI value, the greater the weaning success rate. Figure 3 and Figure 4 present a comparison of DE with RSBI as weaning predictors.

**Table 1:** DE and RSBI parameters and weaning. DE: Diaphragmatic excursion; RSBI: Rapid shallow breathing index.

Parameter	Overall	Successful Weaning (n = 62)	Failed Weaning (n = 28)	P value
DE (cm)	1.44 ± 0.26	1.51 ± 0.26	1.26 ± 0.13	0.0001
RSBI	56.88 ± 8.30	54.05 ± 7.00	63.14 ± 7.64	0.0001

## Discussion

In the ICU, weaning a patient from mechanical ventilation is a vital concern; extubation at the right time avoids weaning failure and mortality [1]. All the patients with difficult weaning history and long periods of intubation have high ICU readmission rates [1]. RSBI is important in predicting the weaning indices presented in a study by Yang and Tobin [3]. RSBI is a collaborative reflection of respiratory mechanics and consists of diaphragm and non-diaphragm muscles. Non-diaphragm inspiratory muscles will compensate if the diaphragm is failing, in order to preserve the tidal volume; diaphragm weakness may be obscured. However, the non-diaphragmatic muscles are more subject to fatigue and are weaker than the diaphragm; they will not be able to provide sufficient ventilation for long [9]. Hence, RSBI may provide false positive extubation criteria, and extubation failure may occur despite an initially adequate tidal volume and good clinical condition for extubation [10,11].

RSBI and DE measurements were taken 20 minutes following the SBT. Also, RSBI conveys the end product of the balance between strength and load on all respiratory muscles. Twenty minutes after the SBT, when all accessory muscles failed to contribute the requisite tidal volume, RSBI accurately indicates whether patients can be successfully extubated. Boussuges et al. reported the DE normal values for deep and quiet breathing were 4.7 and 1 cm, respectively [11]. Lerolle et al. assessed diaphragmatic dysfunction in cardiac patients, reporting a DE less than 2.5 cm might act as prolonged intubation predictor [12]. Hayat et al. concluded that DE plays an important role in weaning; at the cutoff point of 1.2 cm, patients can be successfully extubated [13]. Kim et al. compared the accuracy of DE versus RSBI to predict weaning failure and found they were similar. However, they studied the most difficult patients [14]. Our study indicates an RSBI cutoff of 59 is 79% sensitive and 64% specific for successful extubation. Likewise, a DE cutoff value of 1.35 cm is 74% sensitive and 75% specific. The AUROC of RSBI and DE (0.815 and 0.795, respectively) are significant and comparable. In spite of all documented weaning parameters, some patients may behave totally differently post extubation. Our findings indicate RSBI is a better parameter in predicting weaning outcomes than DE, but DE can be an adjunct parameter with conventional RSBI.

Our study had several limitations in that it was conducted at a single center with variable age group patients population having multiple co-morbids. Our sample size was small; similar studies on a larger scale are warranted to further establish the correlation.

## Conclusions

RSBI and DE are optimized clinical predictors in classifying the weaning outcome in extubation assurance and reintubation prevention. However, further studies are needed to validate the significance of these predictors for weaning.
